# Therapeutic potential of astaxanthin and superoxide dismutase in Alzheimer's disease

**DOI:** 10.1098/rsob.210013

**Published:** 2021-06-30

**Authors:** Vyshnavy Balendra, Sandeep Kumar Singh

**Affiliations:** ^1^ Saint James School of Medicine, Park Ridge, IL, 60068, USA; ^2^ Indian Scientific Education and Technology (ISET) Foundation, Lucknow 226002, India

**Keywords:** oxidative stress, nutraceuticals, astaxanthin, superoxide dismutase (SOD), neurodegeneration, Alzheimer's disease

## Abstract

Oxidative stress, the imbalance of the antioxidant system, results in an accumulation of neurotoxic proteins in Alzheimer's disease (AD). The antioxidant system is composed of exogenous and endogenous antioxidants to maintain homeostasis. Superoxide dismutase (SOD) is an endogenous enzymatic antioxidant that converts superoxide ions to hydrogen peroxide in cells. SOD supplementation in mice prevented cognitive decline in stress-induced cells by reducing lipid peroxidation and maintaining neurogenesis in the hippocampus. Furthermore, SOD decreased expression of BACE1 while reducing plaque burden in the brain. Additionally, Astaxanthin (AST), a potent exogenous carotenoid, scavenges superoxide anion radicals. Mice treated with AST showed slower memory decline and decreased depositions of amyloid-beta (A*β*) and tau protein. Currently, the neuroprotective potential of these supplements has only been examined separately in studies. However, a single antioxidant cannot sufficiently resist oxidative damage to the brain, therefore, a combinatory approach is proposed as a relevant therapy for ameliorating pathological changes in AD.

## Graphical abstract

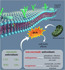

## Research in context

**1. Systematic review.** The authors conducted an electronic search across PubMed, Medline, PsycINFO, ScienceDirect, Google Scholar, Embase library databases for English, peer-reviewed, articles and reviews published after 1964 using the following MeSH terms: Alzheimer's Disease (AD) AND Models, Reactive Oxygen Species (ROS) AND/OR Oxidative Stress (OS) AND Astaxanthin (AS) AND models, Superoxide dismutase (SOD) AND models. Case reports were excluded. The results were further screened by title and abstract for studies performed in rats, mice and humans, at which time full-text articles were screened for eligibility.
**2. Interpretation.** Based on previous studies, an integrated hypothesis is presented, explaining the neuroprotective role of antioxidants in the AD. This proposal provides evidence for the combinatory treatment, of endogenous and exogenous antioxidants simultaneously, for the prevention and treatment of AD.
**3. Future directions.** Future trials should consider administering combinations rather than single antioxidants to facilitate redox cycling as well as maximize bioavailability, efficiency to different cellular compartments and establish the regimens for practical interventions at each stage of AD. The oral bioavailability of AST is limited by its solubility in the gut and lipid-based formulations of AST have been proposed as possible alternatives.

## 1. Objectives

This review aims to synthesize scientific findings on the neuroprotective and therapeutic role of two specific substances—astaxanthin (AST) and superoxide dismutase (SOD)—that each play a role in the endogenous and exogenous pathways of the antioxidant system against oxidative stress (OS). The combination of such substances will be beneficial for patients suffering from Alzheimer's disease (AD).

## 2. Background

Patients with AD, along with their families and caregivers, are faced with devastating health and financial consequences. The number of people living with this disease is projected to increase as the elderly population grows older and life expectancy increases, which will eventually create a strain on the economy [1]. Since OS in the brain increases over time and AD is a significant age-dependent disorder of the brain, the development of novel antioxidant-regulating treatments is crucial for preventing and preserving cognitive function in this population [2,3]. Thus, reducing the levels of ROS is an essential strategy for AD treatment.

### 2.1. Generation of free radicals and oxidative stress

Reactive oxygen species (ROS) are produced by living organisms due to normal cellular metabolism processes like cellular oxidation, cell regulation and signalling [4], and formed by the cells of aerobic organisms such as the electron transport chain, macrophages and peroxisomes [5]. At low to moderate concentrations, ROS can be beneficial and regulate cellular processes such as hormonal regulation and intracellular secondary messaging [6,7]. At higher concentrations, they can adversely modify cellular lipids, protein expression and DNA, eventually causing cell death [8–10]. Free radicals are highly reactive, inorganic and unstable molecules or atoms that have lost one electron, making their outer valance shell incomplete [11]. As a result, these incomplete and intermediate oxygen-carrying metabolites known as oxyradicals aggressively search for their remaining electron in other molecules and, once paired, continue to produce more free radicals [7,12]. In turn, this creates a chain reaction of more unstable free radicals interacting with other molecules leading to more complex and toxic mechanisms [5]. Of these, the most damaging radical in many tissues is the superoxide ion (O_2_^−^) [13], which is produced mainly in the respiratory chain of the mitochondria [14] and is the primary source of all radical-induced toxicity [15,16]. O_2_^−^ is the sequential reduction of molecular oxygen via step-wise addition of electrons, as it can readily produce the hydroxide ion (OH^−^) free radical, which can later cross the cell membrane and cause severe molecular damage known as lipid peroxidation [17]. OH^−^ causes loss of functional integrity of the cell and membrane receptors, alters membrane permeability and increases membrane rigidity, thus decreasing membrane fluidity [18,19]. If the integrity of the cell is not maintained, homeostasis of the cell may be disrupted with little to no chance of reversal. Homeostasis within a biological system is maintained through the balance of antioxidation and oxidation systems [20]. A disruption of this harmony results in a relative deficiency of the antioxidant system, with the favour of ROS, creating an environment known as OS. The persistence of this ongoing rise in ROS molecules leads to adverse modifications in cell components and is marked by lipid peroxidation, high levels of oxidized proteins and oxidative modifications in mitochondrial DNA [21–23]. OS is thought to be involved in the pathophysiology of several chronic diseases like diabetes [7,11,24], macular degeneration [25] and cancer [26,27].

### 2.2. Oxidative stress and Alzheimer's disease

Oxygen is essential for sustaining life, but it does come with a cost as it is also a threat to biological systems. Particularly, the brain accounts for 20% of all the oxygen consumed by the body [28,29] while only weighing 2% of the total body weight [30]. These features make it highly susceptible to oxidative damage due to a high concentration of easily oxidizable polyunsaturated fatty acids, iron and metals, as well as the neuronal metabolic rate which mediates the continuous production of ROS [31]. Neurons are vulnerable to free radicals as there is a rather scarce amount of antioxidant enzymes compared to other organs. For example, catalase (CAT) in the brain is 10–20% of that found in liver and heart [32,33], as well as a high content of methyl ions in specific brain areas [34].

Evidence has consistently supported the involvement of OS as a major contributing factor in physiological ageing and the progression of multiple neurodegenerative pathologies [35–37]. Specifically, though the exact causes and mechanisms underlying AD progression remain unclear and multiple factors have been analysed, OS seems to be a leading contender and has gained importance with the progression of this disease [38,39]. This has been confirmed by the increase in markers of OS such as carbonyls, malondialdehydes and 4-hydroxynonenal [40,41]. Free radical damage in the ageing brain influences A*β* toxicity and tauopathy [42], which are responsible for impairment in memory, thinking and language abilities in AD patients [31,43].

### 2.3. Impact of oxidative stress on neurons

AD is a neurodegenerative disorder characterized by progressive memory loss and disorientation, with extracellular depositions of A*β* protein (senile plaques) and intracellular fibrillary deposits of hyperphosphorylated tau (P-tau) protein (neurofibrillary tangles, NFT). These neuropathological features can eventually lead to neuronal death and brain atrophy [44,45] with the loss of neuronal synapses leading to cognitive impairment [46,47]. A*β* results when there is abnormal cleavage of amyloid precursor protein (APP) by β- and γ-secretases [48,49]. The increase of soluble A*β* creates plaques and a toxic environment to neurons, leading to a decrease in the number and plasticity of synapses [50] and, consequently, initiating the formation of NFT [51]. Accumulation of A*β* causes a loss and malformation of spines due to spine turnover in dendrites [52,53], causing characteristic behavioural and cognitive deficits. It has been demonstrated through cell culture models, transgenic mouse models [21] and post-mortem brains of AD [54] that the abundance of ROS and neuronal oxidation [55–57] activates signalling pathways that alter APP or tau processing [58]. For example, a high concentration of OS stimulates c-Jun amino terminal kinase and p38 MAPK, which increases the expression of β-secretase [59], leading to A*β* deposition [60], while the activation of glycogen synthase kinase 3 (GSK-3*β*) triggers tau phosphorylation and formation of NFT. Also*, in vitro* and *in vivo* studies have shown that OS affects A*β* production and oligomerization, which generates free radicals and, in turn, causes APP processing to create more free radicals, leading to a vicious cycle of OS furthering the neurodegenerative process [61,62].

## 3. Supporting the hypothesis

### 3.1. Antioxidant system: endogenous and exogenous

**Figure 1.**
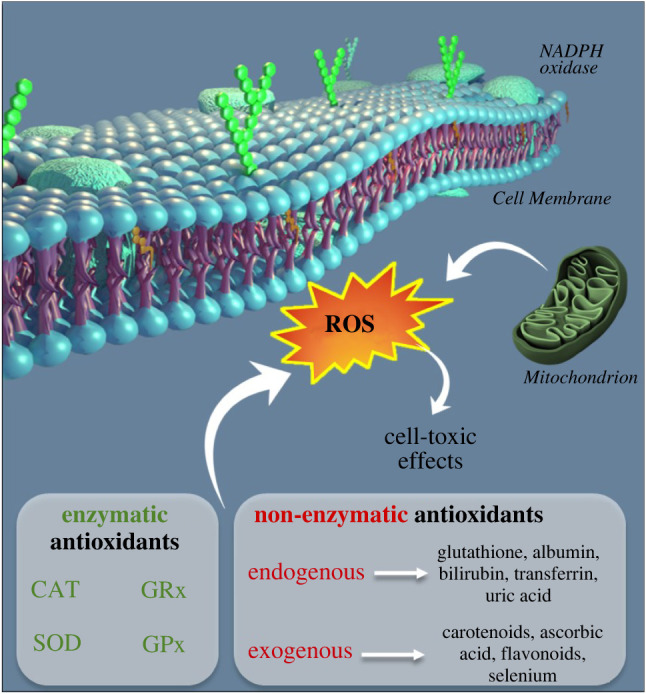
Enzymatic and non-enzymatic classification of antioxidants in the cytosol of cells, acting on the formation of ROS that illicit cell toxic effects.

An ideal, physiologically functioning antioxidant system is a defense mechanism against ROS (figure 1) and is made up of two critical components—the endogenous antioxidants and the exogenous ones. Enzymatic and non-enzymatic endogenous antioxidants include SOD, CAT, glutathione peroxidase (GPX) and glutathione, which are made by the body and are therefore highly potent in repairing free radical damage as they initiate cell regeneration, work in membrane domains and act intracellularly to impact gene expression [64]. Studies have shown that enhanced endogenous antioxidant activity against ROS is directly achieved by the activation of the Nrf2/ARE signalling pathway (nuclear erythroid 2-related factor 2/antioxidant response element) which regulates the expression of these enzymes and is a key mediator in OS [64]. Exogenous antioxidants such as vitamin C, vitamin E, carotenoids and polyphenol are assimilated through diet, undergo a nutritional process and are responsible for repairing free radical damage extracellularly. This can be accomplished at a high kinetic rate by transferring one electron to reduce free radicals [65], quench oxygen singlets, stimulate cell regeneration [66,67] and sequester transition metals through a chelation process [68].

**Figure 2.**
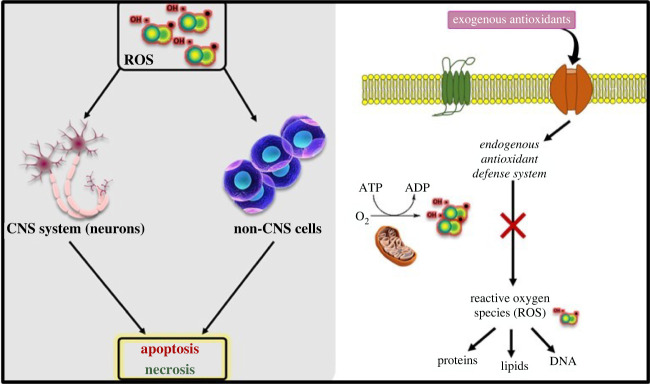
The interplay of exogenous antioxidants and endogenous antioxidants to reduce ROS levels.

Additionally, dietary exogenous antioxidants such as carotenoids and polyphenols impact the proper utility of these enzymatic endogenous antioxidants as they act as enzymatic cofactors [69] in maintaining or re-establishing redox homeostasis in the cell [70]. Studies have demonstrated that there is a potent synergism effect between exogenous and endogenous after revealing that the total moles of radicals neutralized and the velocity by which they reacted to remove free radicals increased due to this combination. This is relevant from a biological perspective given that a faster-marked reaction rate equates to less cellular targets damaged [71]. Thus, dietary exogenous antioxidants play a key role in reinforcing and replenishing the endogenous antioxidant enzymes to eliminate excess oxygen metabolites. Hence, an interactive and often synergistic action occurs between endogenous and exogenous antioxidants to maintain balance with ROS (figure 2) [7,72].

### 3.2. Astaxanthin

AST is a red xanthophyll carotenoid present in freshwater areas and produced by marine microorganisms such as bacteria, yeasts and fungi whereby the richest source is in the microalga *Haematococcus pluvialis* [1,73,74]*.* As a result, AST is consumed by fish such as salmon and trout, giving these organisms a dark red-orange pigment [75,76]. To maintain appreciable levels of AST, it is advised that AST be taken in supplement form (e.g. 4–20 mg), to obtain the beneficial effects rather than through diet alone, which would equate to 600–2000 t of salmon [77]. The unique configuration and size of AST—two hydroxylated ionone rings at both ends of the long carbon chain—allows the molecule to vertically bond to the polar heads of phospholipids and become easily incorporated into the membrane [78]. The shape of AST allows for increased bioavailability and quick absorption into lipids, prevents lipid peroxidation, and increases the stability and integrity of cell membranes [75]. Consequently, after consumption, AST is readily passed through the gastrointestinal (GI) tract into the blood and crosses the blood–brain barrier, eventually treating the brain using AST as a treatment of AD [79–81].

By contrast, comparative studies have shown that AST is 6000 times more potent than vitamin C and 100 times more potent than vitamin E in neutralizing toxic ROS without forming pro-oxidants, commonly known as a negative side effect among many other carotenoids [82,83]. The powerful antioxidant potential of AST is due to its ketone-bearing ionone rings which attract ROS into its polyene backbone. In addition, its ability to freely donate an electron and form chemical bonds with free radicals helps in scavenging free radicals and quenching singlet oxygen particles [84–86]. When a cell is stressed, AST inhibits phosphorylated extracellular regulated protein kinase/extracellular regulated protein kinase ratio (p-ERK/ERK) [87], which increases the concentration of haem oxygenase-1 (HO-1) due to the activation of Nrf2/ARE, consequently promoting the expression of SOD and other endogenous antioxidant enzymes [8,88,89]. These mechanisms of action are of importance given that ROS increases with normal brain ageing [90] and is further increased in AD. Therefore, additional antioxidants need to be in place to keep OS to a minimum.

#### 3.2.1. AST: *in vivo* studies

AST's ability to ameliorate the cognitive decline in normal ageing and decrease the pathophysiology of AD have been investigated by researchers in animal and humans [91,92]. The supplement showed promotion of neuronal survival when differentiated pheochromocytoma (PC12) were subjected to A*β* [93,94] in a mouse model of AD. Extrapolated studies revealed that AST was able to protect neuroblastoma cells from amyloid toxicity by upregulating the HO-1 anti-oxidative enzyme of the haem pathway [95]. Another study demonstrated that the supplement decreased the amount of apoptotic-related mediators such as caspase 3,9, cytochrome C and protected L-glutamate induced cell death in PC12 cells through the Bcl-2/Bax apoptotic pathway [96–98]. These findings were replicated by Lobos *et al.*, and the multipotent nature of the nutraceutical was further shown when primary hippocampal neurons were seen to be protected against amyloid-produced ROS [78]. Densitometry and western blot analysis showed that the expression of P-tau protein was inhibited with the use of AST in transgenic mice [99]. In a recent study, Hongo and colleagues [100] investigated the effects of AST intake on the cognitive and pathological progression of AD. They used AppNl-G-f mice which carry three APP knockout mutations with familial AD causing elevated levels of A*β*42 and observed decreased amyloid deposition, a decline in P-tau-positive areal fraction in the hippocampus (*p* *<* 0.001), increased activation of microglial in the area of amyloid deposition *(p* < 0.001) and a significantly higher mean Parvalbumin-positive (PV^+^) neuron density (*p* = 0.019) in the AST group over the control group. The Barnes maze test was later used to assess memory function in these mice and results indicated a significant difference in the number of visits to the goal region in the control-fed mice as opposed to the AST-fed mice (*p* = 0.0247). In another experiment, the nutraceutical was shown to enrich spatial learning and memory skills by reducing the number of reference memory errors and working memory errors made by APP/PS1 transgenic mice in a radial 8-arm maze apparatus and Morris water maze test *(p* < 0.05) [100]. Overall, AST has a multitude of neuroprotective effects, from reducing oxidative brain dysfunctions and preventing cellular toxicity and neuronal apoptosis to promoting survival in adult hippocampal neurogenesis and improving spatial memory [101,102].

#### 3.2.2. Implication of astaxanthin in clinical studies

**Figure 3.**
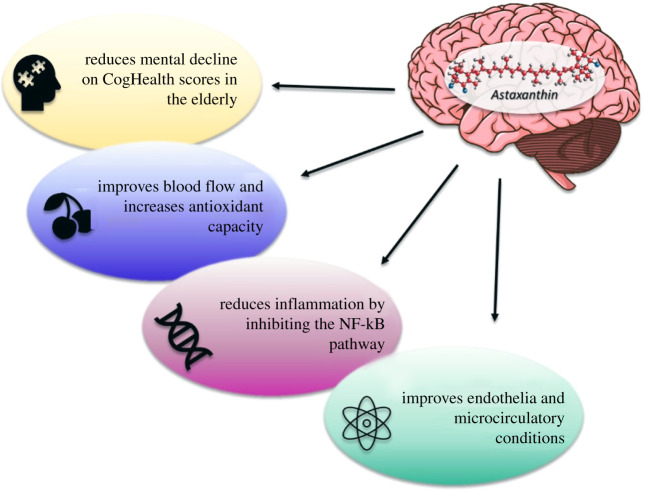
Effects of Astaxanthin on neuronal functions, including the reduction of mental decline in ageing populations, improving blood flow and increasing antioxidant activity, reducing inflammation and inhibiting the NF-kB pathway and improving microcirculatory conditions.

Some preliminary work has begun to examine the therapeutic effects of AST supplementation on humans based on results gathered from *in vivo* trials. A total of 96 healthy subjects ranging from 45 to 64 years of age, with self-reported complaints of age-related forgetfulness, were recruited for a randomized, double-blind, placebo-controlled human trial. It was discovered that AST supplementation, either in the 6 µg d^−1^ or 12 µg d^−1^ group, improved the performance of individuals to a greater degree than the placebo group [103]. Scores in memory and thinking were administered by the CogHealth tests while the executive function was tested using the Groton Maze Learning test, a maze learning paradigm. Repeated trials allowed for the assessment of learning and revealed that the nutraceutical improved cognitive function in aged individuals who had no underlying conditions and participants made fewer errors in the maze test. Subjects who were designated under the high-dose category expressed faster reaction times in the computerized, card-based design test. The efficacy of AST in improving cognitive function was demonstrated in an open-label trial with male participants aged 50–69 with symptoms of mild cognitive deficiencies who were treated with doses of 20 mg d^−1^ for 12 weeks [104]. The CogHealth test battery was used to assess neurocognitive functioning while the P300 was used to measure recognition and selective attention in the context of decision making [105]. Results showed an increase in brain function—cognition, attention, memory, information processing—compared to baseline values (figure 3). Dietary supplementation with AST has been shown to improve psychomotor speed, which is an indication of mental and physical coordination, comprehension and ability to perform complex tasks efficiently and accurately [106,107].

Elevated lipid oxidation [108,109] and phospholipid hydroperoxide (PLOOH) are present in abnormally high levels in erythrocytes of AD patients. However, in a double-blind human trial with participants 50–69 years of age, it was noted that after a 12-week AST diet, plasma and erythrocyte concentrations of PLOOH decreased compared to the control group, which confirms that AST improves antioxidant levels in cells [110]. OS influences the pathogenesis of neuronal loss in neurodegenerative diseases, especially AD, and, as a result, the neuroprotective capability of this substance is of value for co-treatment in the prevention of these diseases [111].

### 3.3. Superoxide dismutase

As previously mentioned, O_2_^−^ is the most common ROS [26,112]. SOD is a metalloenzyme that forms the first-line antioxidant defense mechanism and is one of the major enzymatic components to detoxify superoxide radicals [113]. ROS production is a cascade effect that initially begins with the production of O_2_^−^ but can be neutralized by SOD as means of protecting the cells [114]. This renders the potentially harmful O_2_^−^ less hazardous [115–117]. By removing O_2_^−^, SODs decrease the risk of OH^•^ formation via the Haber–Weiss-type reaction which has a 10 000-fold faster rate than spontaneous dismutation [119] and instead, reacts in the presence of Fe^2+^ through the Fenton reaction to form hydrogen peroxide (H_2_O_2_) and oxygen (O_2_). This enzyme is unique in that its activity determines the concentrations of O_2_^−^ and H_2_O_2_, the two Haber–Weiss reaction substrates, and is therefore likely to be central in the antioxidant defence mechanism [118,120]. SOD can eliminate O_2_^−^ rapidly due to its ability to convert a second-order reaction to a first-order reaction [121,122]. However, if the concentration of free radicals overwhelms the capacity of the enzyme, the O_2_^−^ can combine with NO to form peroxy-nitrite or undergo the Fenton reaction to form OH^•^ radicals [123], which is a stronger oxidant and far more damaging than O_2_^−^.

SOD consists of three types of isoforms that are found in mammalian cells: copper/zinc SOD (CuZn-SOD), which is a cytoplasmic enzyme; manganese SOD (Mn-SOD, SOD_2_), which is a mitochondrial matrix enzyme; and extracellular SOD (EC-SOD, SOD_3_) [124]. Converging evidence confirms that most of the proteins associated in the pathogenesis of AD have direct involvement with mitochondrial enzyme SOD–MnSOD [125]. Observations from studies have shown that SOD knockout mice accelerate A*β* plaque deposition [126], increase tau phosphorylation [127] and worsen behavioural deficits [128], all suggesting that SOD plays a pivotal role in human ageing and AD. Unfortunately, it has been found that the SOD molecule is deactivated and does not become bioavailable as it passes through the GI tract once it encounters acids and enzymes [129,130]. As a result, scientists have worked around this problem by having SOD coupled with a protective protein derived from wheat, which can then sustain the gastric acids and be delivered in full form and absorbed into the bloodstream, thus effectively enhancing the body's own primary defence system [131,132]. Research has shown that oral SOD is linked with the induction of endogenous antioxidant enzymes' expression of CAT, GPX and further SOD [133,134]. These specific enzymes decrease in concentration and the production of free radicals increases with age [135], and it has been shown that this supplement could increase and strengthen the body's endogenous supply, increase the activities of all three enzymes and establish a reduction in neuronal damage in AD patients.

#### 3.3.1. SOD: *In vivo* studies

The most abundant ROS in cells influencing synaptic plasticity, memory function and neuronal death is the superoxide radical [136]. SOD plays a protective role in neurodegeneration and has been shown to protect the ageing brain against human APP (hAPP)/Aβ-induced impairments after learning that inactivating one SOD allele in human hAPP transgenic mice led to a depletion of microtubule-associated protein 2 (neuronal dendritic marker) in the hippocampus and neocortex, decreased astrocytosis, promoted cerebrovascular amyloid gliosis and plaque-dependent neuritic dystrophy. In regard to behavioural issues, the lack of one SOD allele led to alterations in behaviour with lower anxiety levels and reduced disinhibition. Previous work conducted on mice with SOD deficiency in Tg2576 AD hastened the process of A*β* aggregation [137]. Other studies revealed that mice overexpressing SOD showed a reduction in plaque production and less memory impairments [138]. SOD supplementation was able to contrast the observed exacerbation of all AD-like features as well as counteract the manifestation of cognitive impairments by effectively responding to oxidative injuries [139,140]. OS affects A*β*PP processing at least in part by upregulating β-site A*β*PP cleavage enzyme BACE1 (beta-site APP cleaving enzyme 1) [141]. SOD treatment significantly downregulated the BACE1 enzyme in Tg19959 mice and brain levels of A*β* were decreased. SOD mimetics given to ageing mice for 6 months resulted in decreased lipid peroxidation, nucleic oxidation and ROS levels, and improved age-related decline in performance during fear conditioning tasks [142]. This study showed that age-dependent OS increases resulted in learning and memory deficits due to damage to the hippocampus and amygdala and could be significantly reduced by chronic treatment of SOD, indicating that it is oxidative load in aged mice that accounts for 50–60% of the variance in learning ability [56,143]. Antioxidant treatment, especially EUK-207, which is a SOD mimetic, inhibited the progression of tau phosphorylation and, in effect, decreased the disease symptoms in 3X-Tg-AD, an aggressive mouse model of AD [144].

#### 3.3.2. Implication of SOD in clinical studies

Information from animal studies has been applied to clinical studies and similar outcomes have been seen in humans. A randomized clinical study of 20 healthy volunteers was exposed to pure oxygen (2.5 absolute atmospheres) for 60 min to induce cellular OS. They were then orally treated with Gliosidin (SOD supplement) and their SOD enzymatic activities were analysed in erythrocytes. It was found that SOD played a critical role in protecting DNA from cellular damage due to hyperbaric oxygen [131]. A double-blind, placebo-controlled pilot study was conducted using the melon juice concentrate, 10 mg Extramel (140 IU SOD per capsule) in 70 healthy volunteers between the ages of 30 and 55 years (mean = 40.26 years) with a BMI from 17 to 42, who felt daily stress and fatigue. Participants reported a reduction of stress and physical fatigue, as well as a significant improvement in cognitive performance on psychometric scales: Ferreri Anxiety Rating Diagram (*p* = 0.032), Cohen Perceived Stress scale (*p* = 0.01), 12-Item Short Form Survey (*p* = 0.049) after four weeks of oral supplementation [145]. A similar study was carried out by Carillon *et al.* [133], showing that three months of Extramel treatment improved the score on the Stroop test (27.9%; *p* < 0.001) which is used to measure cognitive flexibility, processing speed and overall executive function.

## 4. Testing the hypothesis

Oxidative damage is not just a by-product or end product of AD but is the direct initiation in the process of neurodegeneration [146]. Accumulation of amyloid in AD changes the expression of antioxidant genes, which further adds to the oxidative damage, free radicals and neuronal dysfunction [147]. Although the aetiology of AD is multifactorial, the combination of genetic and environmental factors, which includes nutrition, plays a central role in the onset and progression of the disease. Nutritional intervention can present a relevant route to achieve beneficial effects in AD treatment, at least in combination with pharmaceutical therapy. The administration of exogenous antioxidants is beneficial in treating the side effects of OS by compensating the inefficacy of the endogenous defense systems through inhibiting the intricate network of oxidative damage pathways and enhancing the systemic antioxidant response [20]. AST acts through exogenous antioxidant mechanisms as well as stimulating endogenous anti-oxidative enzymes [148,149,153], which is of importance given that, with age, the concentration of endogenous antioxidant enzymes decreases along with the efficiency and activity of these enzymes. Previous studies have shown that oral supplementation of SOD was found to promote the circulation, in blood and brain, of endogenous enzymes such as SOD and CAT [154]. This promoting effect will go on to combat the OS occurring in the cell and serve as neuroprotection. Substantial evidence discussed above suggests that the two nutraceutical molecules, AST and SOD, can synergistically work together as each plays a role in the exogenous and endogenous antioxidant system. Thus, it is predicted that AST and SOD will exhibit stronger anti-oxidative activity and provide pleiotropic functions than AST or SOD alone in improving learning and memory abilities in different degrees across AD patients. However, to date no experimental trials have been conducted to verify this dual combination. Future trials should consider administering combinations rather than single antioxidants to facilitate redox cycling as well as maximize bioavailability efficiency to different cellular compartments and establish the regimens for practical interventions at each stage of AD.

### 4.1. Limitations

The need to find and develop innovative delivery systems is of necessity when it comes to AST, due to its low bioavailability, poor water solubility and susceptibility to heat stress [150–152]. There have been some promising suggestions to address this drawback and, recently, AST has been formulated with lipid-based carriers such as oil-loaded solid lipid nanoparticles, constructed lipid carriers and cyclodextrin. These novel delivery systems have been shown to increase its stability and thus potentiate its antioxidant capacity [150,155,156]. Unfortunately, more experimentation is required to develop an appropriate and potent delivery system to precisely study AST's multi-target neuroprotective effects [157]. Other challenges include developing standardized, precise and definitive biomarkers of OS that can be used as early detection for AD [158,159]. From there, experiments need to be examined to validate if a causal relationship exists and whether those markers respond to antioxidant intervention [160]. Further studies should be conducted in order to determine whether the simultaneous ingestion of nutraceuticals contributes to an overall practical and beneficial healthcare strategy in the treatment of AD. Importantly, an optimum combination of SOD and AST in terms of doses is an area that still needs to be analysed, as the removal of many ROS by supplementation of antioxidants may cause ‘anti-oxidative stress’, which can be detrimental to neuron physiology by disrupting cell signalling pathways in the brain and worsening the disease [161–163].

### 4.2. Translational concerns

Despite clear implications that oxidative damage is a key factor in the pathophysiology of AD and literature suggesting the therapeutic nature of AST and SOD in targeting ROS/antioxidant imbalance, there seems to be a translational problem. Improper design of human intervention studies [164,165] such as a low number of recruited participants [145], short duration of treatment and analysis of end points, which are related to pharmacokinetic and pharmacodynamic constraints, can all prevent the possibility to detect potential improvements in cognitive function in these patients. The lack of efficient animal models to mimic the overall pathophysiological conditions in the pathogenesis of AD in humans could also be a reason for low clinical applications [166]. There is also a concern to determine different doses depending on gender, age, underlying health issues in accordance with AD, previous drug use, social habits, baseline nutritional levels of patients, genotype and biochemical status, which could explain inter-individual differences in terms of bioavailability [167]. Decreased rates of success in clinical trials could be the result of supplements being administered in patients with advanced stages of AD, while most of the *in vivo* studies have been performed at earlier stages, and therefore a very large time gap exists, possibly decades, from pre-clinical signs to the clinical onset of AD [168,169]. Therefore, these supplements should be tested in earlier phases of the disease to uncover their therapeutic potential. Lastly, the most crucial factor is the brain's complexity. OS causes neuronal dysfunction inducing compensatory responses and a change in neural circuitry [170]. This scenario complicates the efficacy of antioxidant therapy due to the regeneration of the lost neuronal network that is found in the ageing brain.

## 5. Conclusion

The prevalence and high mortality of AD presents medical and financial burdens on society, specifically the patients' caregivers. An important feature of ageing is the weakening of the biological antioxidant system defence system such as the loss of endogenous antioxidant enzymes like SOD, as well as the increasing levels of ROS, creating a state of OS in the brain. Many AD histopathological studies confirm disruption of the redox homeostasis in the brain as playing a fundamental role in amyloid plaque formation and hyperphosphorylation of tau protein [171], which contribute to impairments in cognition [172]. Focusing on the development and utilization of antioxidant therapies can assist in reducing toxic depositions. Various clinical and basic research studies have provided support for antioxidant treatment in AD, and it is likely that a single antioxidant may not be sufficiently resistant to oxidative damage given that OS is modulated by a complex system of endogenous and exogenous antioxidants [78]. The integrated approach of AST and SOD antioxidant therapy, along with first-line synthetic drugs, is suggested to provide a promising natural treatment alternative in delaying the progression of AD. Future research can elucidate the role of both these compounds, which can provide insight into providing safe and potent neuroprotective agents that could improve the quality of life and life expectancy of these patients.

## Data accessibility

This article does not contain any additional data.
